# Metabolic Profiling of *IDH* Mutation and Malignant Progression in Infiltrating Glioma

**DOI:** 10.1038/srep44792

**Published:** 2017-03-22

**Authors:** Llewellyn E. Jalbert, Adam Elkhaled, Joanna J. Phillips, Evan Neill, Aurelia Williams, Jason C. Crane, Marram P. Olson, Annette M. Molinaro, Mitchel S. Berger, John Kurhanewicz, Sabrina M. Ronen, Susan M. Chang, Sarah J. Nelson

**Affiliations:** 1 Department of Radiology & Biomedical Imaging, University of California, San Francisco, CA, USA; 2 Department of Bioengineering and Therapeutic Sciences, University of California, San Francisco, CA, USA; 3 Department of Pathology, University of California, San Francisco, CA, USA; 4 Department of Neurological Surgery, University of California, San Francisco, CA, USA; 5 Department of Biostatistics and Epidemiology, University of California, San Francisco, CA, USA.

## Abstract

Infiltrating low grade gliomas (LGGs) are heterogeneous in their behavior and the strategies used for clinical management are highly variable. A key factor in clinical decision-making is that patients with mutations in the *isocitrate dehydrogenase 1* and *2 (IDH1/2*) oncogenes are more likely to have a favorable outcome and be sensitive to treatment. Because of their relatively long overall median survival, more aggressive treatments are typically reserved for patients that have undergone malignant progression (MP) to an anaplastic glioma or secondary glioblastoma (GBM). In the current study, *ex vivo* metabolic profiles of image-guided tissue samples obtained from patients with newly diagnosed and recurrent LGG were investigated using proton high-resolution magic angle spinning spectroscopy (^1^H HR-MAS). Distinct spectral profiles were observed for lesions with *IDH-*mutated genotypes, between astrocytoma and oligodendroglioma histologies, as well as for tumors that had undergone MP. Levels of 2-hydroxyglutarate (2HG) were correlated with increased mitotic activity, axonal disruption, vascular neoplasia, and with several brain metabolites including the choline species, glutamate, glutathione, and GABA. The information obtained in this study may be used to develop strategies for *in vivo* characterization of infiltrative glioma, in order to improve disease stratification and to assist in monitoring response to therapy.

Infiltrating gliomas comprise 80% of malignant brain tumors and consist primarily of astrocyte and oligodendrocyte cell types. Tumor grade is assessed according to criteria set by the World Health Organization (WHO)^1^. Patients diagnosed with Grade II glioma are considered to be low grade (LGG), and may survive for years or even decades^2^,^3^. In making decisions about clinical management, it is necessary to strike a balance between treating the tumor and impacting quality of life. More aggressive treatments are often reserved for tumor recurrence, when lesions may undergo malignant progression (MP) to Grade III (anaplastic glioma) or Grade IV glioma (secondary GBM)^4^. Unfortunately, at present, there is a significant lack of diagnostic and prognostic biomarkers that are able to non-invasively assess MP and predict clinical outcome.

In 2009, Yan *et al*. discovered novel missense mutations in the *isocitrate dehydrogenase 1 & 2 (IDH1/2)* oncogenes, which are present in upwards of 70–80% of lesions that arise from low-grade lineage and confer a significant survival advantage over wild-type tumors^5^. Interestingly, *IDH1/2* mutations were found to be conserved across mutually exclusive pathways of *TP53* mutation and 1p19q chromosomal loss and have been implicated as the earliest known mutation event in gliomagenesis^6^. Given the limited therapeutic options available for LGGs, *IDH* mutations have garnered significant attention as a targetable therapeutic pathway with several novel therapies currently in development and under investigation in clinical trials^7^,^8^,^9^.

The *IDH1/2* genes natively encode the isocitrate dehydrogenase (IDH) enzymes required for the decarboxylation of isocitrate to α-ketoglutarate in the cytosol and mitochondria, respectively. The heterozygous nature of *IDH* mutations results in an amino-acid substitution at one of two enzymatic active sites, most commonly arginine to histidine, creating a wild-type and mutant heterodimer. The wild-type position continues the production of α-ketoglutarate, while the mutant portion develops the gain-of-function ability to further convert α-ketoglutarate to D-2-hydroxyglutarate (2HG), an otherwise scarce metabolite in normal cells^10^. 2HG accumulates up to millimolar concentrations in *IDH*-mutant tumors and drives modification to the epigenetic landscape and cellular processes within the tumor^11^.

High-resolution Magic Angle Spinning (HR-MAS) is a powerful spectroscopic technique for interrogating the biochemical properties of tissue^12^. While 2HG is readily detectable in an *ex vivo* setting using HR-MAS, it has proved to be a difficult biomarker *in vivo* owing to low signal-to-noise and significant overlap with neighboring metabolites at clinical field strengths. Our group and others have shown that metabolic differences can differentiate between cancer types and histological grades^13^,^14^,^15^. Here, we further hypothesized that there are differences in metabolite levels for lesions that contain the *IDH*-mutated genotype, as well as those that have undergone MP.

The objective of this study was to characterize the *ex vivo* metabolic profiles from patients with a range of infiltrating glioma. The observed profiles were related to findings from histological analysis of sampled tissue with respect to variations in *IDH* mutation status, histological subtype, and tumor grade. The long-term goal is to use the information obtained as a means to design *in vivo* metabolic imaging methods that could improve the characterization of this disease, and provide biomarkers for non-invasive monitoring of disease progression and malignant progression in patients.

## Methods

### Patient population

The study was approved by the Institutional Review Board (IRB) at the University of California, San Francisco (UCSF). Patient accrual was performed prospectively and informed consent was obtained from each participating subject, with all experiments carried out in accordance to the protocols set by the IRB. A total of one hundred and twenty-six patients were included. Thirty-six of the patients had newly diagnosed, non-enhancing lesions. Ninety patients had an initial diagnosis of WHO grade II glioma and were presenting for surgical resection owing to suspected recurrence, when MP is commonly observed.

### Presurgical *in vivo* MR imaging and spectroscopy

MR examinations were performed on either a 1.5 T or 3 T MR scanner (GE Healthcare Technologies) using an eight-channel phased-array head coil (MRI Devices). Standard anatomical imaging included T_2_-weighted (FLuid Attenuated Inversion Recovery (FLAIR) and Fast Spin Echo (FSE)) as well as T_1_-weighted pre- and post-gadolinium contrast images. Diffusion Weighted Imaging (DWI) was obtained in the axial plane with 6 gradient directions and two-fold acceleration with sensitivity encoding parallel imaging [repetition time (TR)/echo time (TE) = 1000/108 ms, voxel size = 1.7 × 1.7 × 3 mm^3^, b = 1000 s/mm^2^]. Dynamic Susceptibility Contrast (DSC) Perfusion Weighted Imaging (PWI) was obtained with a 5 ml/s bolus injection of 0.1 mmol/kg body weight gadolinium diethyltriamine pentaacetic acid (Gd-DTPA) and acquired using a series of T_2_*-weighted echo-planar images [TR/TE/Flip-angle = 1250–1500/35–54 ms/30–35 degrees, 128 × 128 matrix, slice thickness = 3–5 mm, 7–15 slices with 60–80 time points] before, during, and after injection. Lactate-edited 3D proton MR spectroscopic imaging (MRSI) was acquired using point-resolved spectroscopic selection (PRESS) for volume localization and very selective saturation (VSS) pulses for lipid signal suppression [approximate excited volume = 80 × 80 × 40 mm^3^, TR/TE = 1104/144 ms, overpress factor = 1.5, field of view = 16 × 16 × 16 cm^3^, nominal voxel size = 1 × 1 × 1 cm^3^, flyback echo-planar readout gradient in the SI direction, 988 Hz sweep width and 712 dwell points]^15^.

### Post processing of MR data

The MR data were de-identified and transferred to a local Linux workstation. Software developed in-house was applied to estimate relevant DWI, PWI, and MRSI parameters and to normalize between field strengths and subjects using estimates from Normal Appearing Brain Tissue (NABT). Maps of the normalized Apparent Diffusion Coefficient (ADC) were generated on a voxel-by-voxel basis according to a published algorithm^16^. Perfusion datasets were non-rigidly aligned using the VTK CISG software package^17^. Normalized Cerebral blood volume (CBV), percent ΔR2* signal recovery (%-REC), ΔR2* peak height (PH), and recirculation factor were calculated for each voxel using software developed by our lab. CBV intensities and PH parameters were obtained by fitting the dynamic perfusion data by a modified gamma-variate function with a recirculation parameter^18^. Peak height and percent recovery values were also estimated using a simplified nonparametric procedure^19^.

Lactate-edited MRSI data were reconstructed and the signal from the individual channels combined to quantify total choline (tCho), N-acetyl-aspartate (NAA), creatine (Cr), lactate (Lac) and lipid (Lip) levels. The choline-to-N-acetyl-aspartate index (CNI) was generated from an iterative linear regression based algorithm^20^ and represents changes in choline and NAA levels relative to voxels in NABT. Imaging data were aligned to the post-gadolinium T_1_-weighted images using FMRIB’s Linear Image Registration Tool (FLIRT). Anatomic imaging was resampled for overlay with DWI, PWI, and MRSI and the SIVIC software package was used to select target locations for intra-operative tissue sampling^21^.

### Tissue sample acquisition

Tissue sample targets were planned for each patient based on surgically accessible regions with abnormally decreased ADC, decreased %-REC, increased CBV and elevated CNI. These were expected to represent viable regions of tumor with elevated proliferation and neovascularization. The target locations were designated as 5-mm-diameter spheres on co-registered MR images and transferred to the surgical navigation workstation (BrainLAB Inc.). The participating neurosurgeons were guided to these designated locations and tissue samples were excised if it was possible to do so safely. Samples were immediately bisected, with half being fixed in 10% zinc formalin, dehydrated by graded ethanols, and embedded in Paraplast Plus wax (McCormick Scientific) using standardized techniques for tissue processing and immunohistochemistry. The other half was snap-frozen in liquid nitrogen and stored at −80 °C for analysis with ^1^H HR-MAS.

### Histopathology and IDH-analysis

Tissue samples were reviewed and scored for standard WHO criteria by a board-certified neuropathologist. Antibodies used in the assessment of the samples included rabbit polyclonal MIB-1 anti-Ki67 (30-9) (Ventana Medical Systems) at 2 μg/ml for 23 min at 37 °C; mouse anti-SMI-31 (Covance) at 1.5 μg/ml for 8 min at 37 °C; rabbit polyclonal Factor VIII (Dako) at 1.2 μg/ml for 20 min at 37 °C; and mouse monoclonal anti-*IDH1*R132H (DIA H09) (Dianova) at 1:50 μg for 32 min at 37 °C^23^. Heat antigen retrieval for MIB-1 was performed for 30 min in citrate buffer at pH 6. *IDH1*R132H and Factor VIII staining was performed in Tris-EDTA buffer at pH 8. Following antigen retrieval, sections were treated with 3% methanol-hydrogen peroxide for 16 min at 22 °C. All immunohistochemistry assays were performed on the Ventana Medical Systems Benchmark XT. Additionally histopathological methodology can be found in the Supplemental Materials.

### *Ex vivo* HR-MAS spectroscopy

Image-guided tissue samples were transferred to a pre-chilled environment and loaded into a chilled 35-ml zirconia rotor (custom-designed by Varian) with 3 ml of 99.9% atom-D deuterium oxide containing 0.75 wt% 3-(trimethylsilyl) propionic acid (Sigma-Aldrich) for chemical shift referencing. Data were acquired at 11.7 T, 1 °C, 2250 Hz spin rate in a 4-mm gHX nanoprobe with a Varian INOVA 500 MHz multinuclear spectrometer. The nanoprobe gHX is an inverse probe, optimized for the direct detection of protons and the indirect detection of X-nuclei (^13^C, ^31^P, ^15^N) and was equipped with a magic angle gradient coil. A rotor-synchronized T2-weighted Carr-Purcell-Meiboom-Gill (CPMG)^22^ pulse sequence was chosen for its ability to eliminate broad macromolecular signals, and run with a TR/TE = (4 s)/(144 ms), 512 scans, 40,000 acquired points, 90° pulse, and 20 kHz spectral width for a total time of 35 min.

Preprocessing of HR-MAS spectra was done in the time domain using the Java-based magnetic resonance user interface (jMRUI)^23^. All data was normalized by tissue sample weight to correct for differences in signal intensity. Quantification of metabolite levels was achieved with the semi-parametric algorithm HR-QUEST, which fits a customized basis set of metabolites to a given spectrum^24^. The HR-QUEST basis set used in this study comprised spectra from 27 metabolites that are commonly studied in human brain tumors. Only metabolite levels with less than 13% Cramer-Rao error estimates were included in the analysis. Average spectral profiles were produced using in house algorithms that frequency-shifted and normalized each post-processed spectrum by tissue size. Two experienced spectroscopists evaluated each spectrum to qualitatively assess goodness of metabolite fits, the presence of 2HG levels, and whether low resolution or signal-to-noise compromised its analysis. Patients were treated as entirely *IDH* mutated if 2HG was determined to be present in any tissue sample spectrum or if either immunohistochemistry for *IDH1*^*R132H*^ or Sanger sequencing for other variants of *IDH1/2* were positive. This combined approach reduced the risk of false-negatives based on the other mutational variants of *IDH1* and *IDH2*, which would not be detected under IHC alone.

### Heatmap generation

A spectral heatmap was generated from the HR-MAS data using the following procedure: each metabolite was normalized by the 90^th^-percentile for relative visualization, the tissue samples were categorized by grade, the data were imported into Gitools version 2.2.1 (www.gitools.org)^25^ and a linear, hierarchical clustering algorithm was performed at the tissue sample level.

### Statistical Analysis

All statistical testing was performed in R (version 3.1.2). Mixed effect logistic regression modeling that incorporated the effect of multiple tissue samples per patient was used to determine the significant differences in metabolite levels between different tumor grades and *IDH* mutation status. Measured levels of each metabolite were used as lone predictors for a binary outcome in the model. Odds ratios and Wald statistic *p* values were reported together with a 95% confidence interval for each metabolite and group comparison. Statistical significance was assessed at *p* < 0.05.

The Pearson product-moment correlation test was used to assess pair-wise correlations between 2HG levels and other continuous variables, and for the subset of ordinal variables, a Kendell tau rank correlation was performed. In order to address the multiplicity of observations in each biopsy event, each correlation was run fifty times with a random selection of one sample per event. The mean and standard deviation of the Tau estimates were reported with the median *p* value of all tests. To assess confounding relationships with 2HG levels and differences in tumor cellularity, this process was repeated after normalizing metabolite levels by cell density as determined by the average number of cells per 200x field. For 2HG and metabolite level correlations, a Bonferroni alpha-correction was used as a conservative threshold of significance at *p < *0.002.

## Results

### Characterization of the patient population

A summary of the patient population is presented in Table 1. The majority of the patients (108 patients, 88%) were found to harbor *IDH* mutations. These lesions were distributed across all histological subtypes, including 52 patients with astrocytoma (AS), 44 patients with oligodendroglioma (OD), and 30 patients with mixed oligoastrocytoma (OA). There were 57 tissue samples from 36 patients who had non-enhancing, newly diagnosed WHO Grade II (25 patients, 38 samples), WHO Grade III (9 patients, 15 samples), or WHO Grade IV glioma (2 patients, 4 samples). There were 162 tissue samples from 90 patients with an initial diagnosis of LGG who presented at the time of suspected disease recurrence, with a median time to recurrence of 6.1 years. Fifty-one percent of the population was determined to be Grade III or Grade IV and forty-nine percent was Grade II. Of the 65 patients where clinical 1p19q chromosomal data was available, 36 were co-deleted, 27 remained intact, and 2 patients had deletion of the 19q arm only.

### Metabolic differences associated with *IDH* mutation and MP

**Figure 1:**
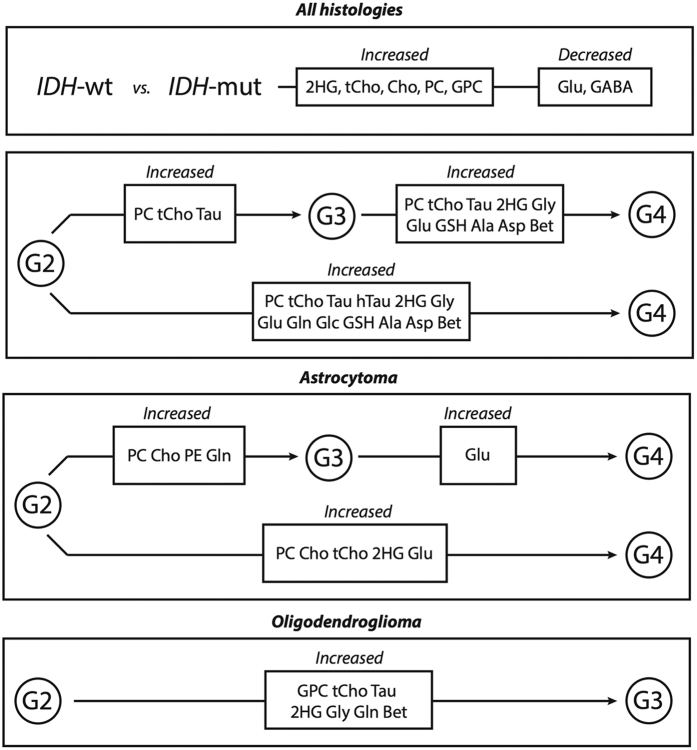
Flow diagram of metabolite differences associated with *IDH* mutation status and MP. Here we present the metabolic differences associated with *IDH* mutation versus wild-type lesions, as well as profile differences associated with malignant progression across all histologies, as well as within individual astrocytoma and oligodendroglioma subtypes. Within *IDH*-mutated lesions, we found significant elevations in 2HG, and found decreases in Glu and GABA versus wild-type. When assessing Grade II and III lesions, we found increases in the choline species associated with the *IDH* genotype. Further, we found several metabolite levels to be increased in tumors that had undergone MP versus those that did not, including specific differences associated with MP within distinct histological subtypes.

**Figure 2:**
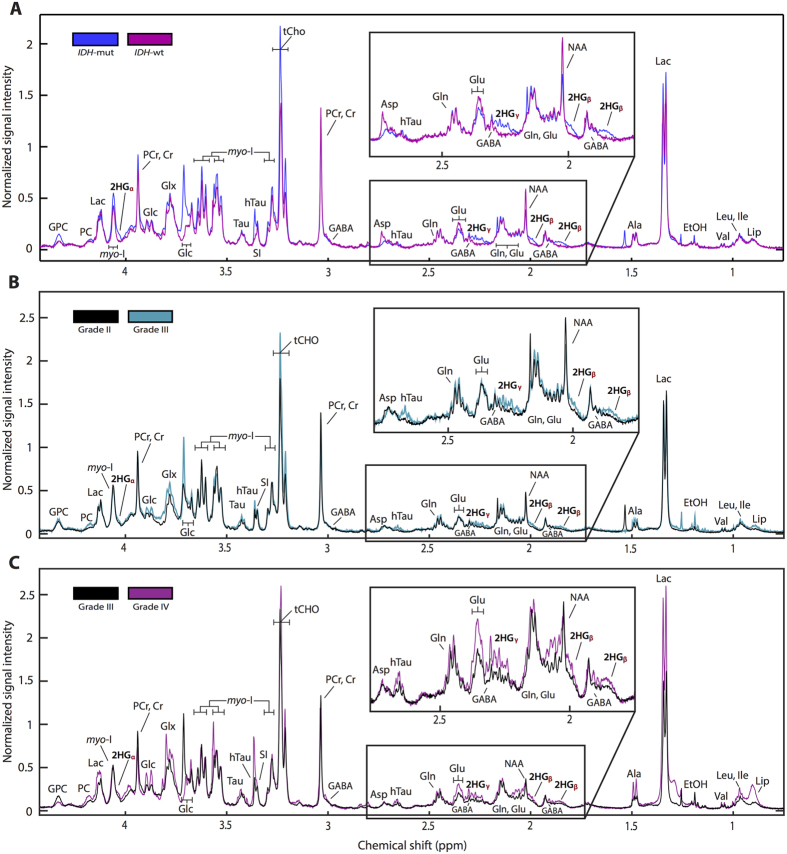
Averaged HR-MAS spectral profile from *IDH* mutation status and MP across different histological grades. Results were normalized by tissue weight to produce an averaged spectrum at the tissue sample level for *IDH* genotypes and individual grades. *IDH-*mutated spectral profiles demonstrate increased 2HG, Cho, GPC, PC, and tCho and decreased Glu and GABA (**A**), while many metabolites were elevated in tumors that had undergone MP (**B**,**C**).

**Figure 3:**
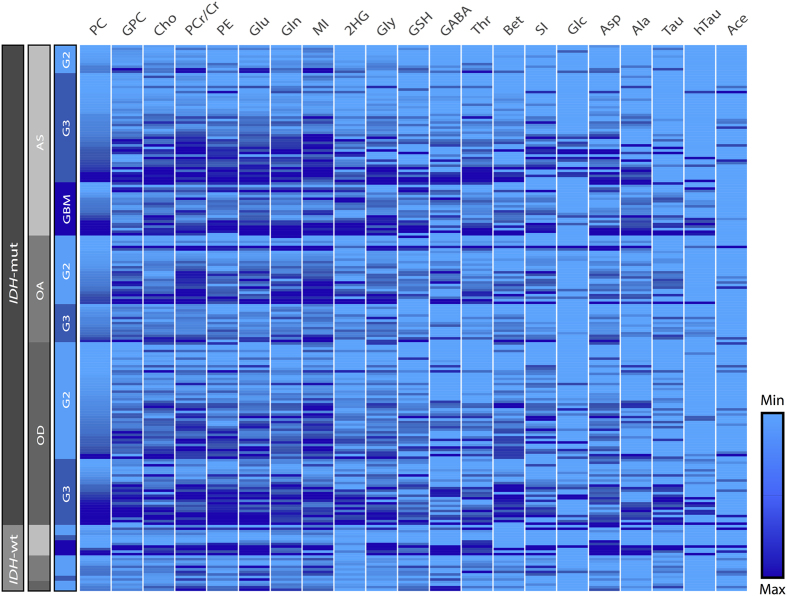
Metabolic spectral heatmap across samples categorized by *IDH* mutation, histological subtype, and grade at the time of surgery. The heatmap was generated from the HR-QUEST quantification of individual tissue sample spectra (rows) and organized by metabolite (columns). Data were normalized by the 90^th^-percentile across columns and sorted by PC within each grade providing comprehensive visualization of the entire dataset and distribution of metabolite levels across subtype and histological grades. We observed intra-patient heterogeneity of tissue samples, as well as global elevation in the key metabolites associated with *IDH* mutation, histological subtype, and MP.

There were significant metabolic differences in *IDH-*mutated lesions when compared to wild-type. Within all grades, *IDH*-mutated lesions were found to have increased levels of 2HG and decreased levels of Glu and GABA compared to wild-type lesions (Table 2). Within Grade II and Grade III lesions, there were elevations of free choline (Cho), glycerophosphocholine (GPC), PC, and the combined measure of tCho in *IDH*-mutated lesions. Addditionally, we found significant elevations of several metabolites in samples from lesions that had undergone MP. The magnitude of the observed differences varied based on tumor grade and histological subtype as presented in Fig. 1 and Table 2. Figure 2 presents comparisons of averaged spectra based on *IDH* status (plot A), as well as upon the grade of glioma at the time of surgery (plots B and C). Lesions that had undergone MP displayed statistically significant elevations of phosphocholine (PC) and total choline (tCho). Elevated levels of taurine (Tau) were associated with secondary Grade III and Grade IV lesions, and elevated levels of hypo-taurine (hTau) with secondary Grade IV lesions. Increased glycine (Gly), glutamate (Glu), glutathione (GSH), alanine (Ala), aspartate (Asp), and betaine (Bet) were found to be significantly elevated in lesions that had undergone MP to Grade IV compared with all other grades, while glutamine (Gln) and glucose (Glc) were elevated in secondary Grade IV tumors relative to Grade II lesions. 2HG levels were elevated in secondary Grade IV lesions across all histologies. A spectral heatmap produced from the dataset is presented in Fig. 3, and displays normalized spectral intensities across all tissue samples. Intra-grade clustering provided visualization of the entire population and distribution of metabolite levels in individual samples.

When assessed by AS or OD histological subtype, there were distinct differences in MP for each tumor type, which are presented in Supplementary Table S1. Anaplastic ASs displayed marked elevations of PC and Cho, while anaplastic ODs displayed elevations of GPC. In AS lesions, increased phosphoethanolamine (PE) and Gln were observed in Grade III versus Grade II samples, while elevated glutamate was observed in secondary Grade IV samples. 2HG was found to be elevated in secondary Grade IV lesions and Grade III oligodendrogliomas. Other metabolites that were increased in anaplastic OD included Tau, Gly, Gln, and Bet. Supplementary Figure S1 presents differences between AS and OD histological types for samples from Grade II and III lesions. In OD tumors, there was elevated PC and Cho in Grade II samples, increased 2HG and GPC in Grade III samples, and elevated Eth when Grade II and III samples were combined. Mixed oligoastrocytomas were excluded from histological subtype analysis due to the uncertainty in how to classify them in the absence of 1p and 19q chromosomal information.

### Correlation of 2HG with metabolite levels and histopathology parameters

Given that the study population was predominately *IDH*-mutated, this provided a unique opportunity to investigate correlations of 2HG levels with other brain tumor metabolites and histopathology parameters in these tumors. These results are presented in Table 3. There were positive correlations of 2HG levels with all of the choline-containing compounds (Cho, GPC, PC, and tCho), PE, GSH, Tau, Glu, Gln, Asp, myo-I, SI, GABA, PCr/Cr, Gly, Bet, and Thr. In addition, there were correlations with 2HG levels and mitotic activity, as measured by MIB1 antibody staining; increased axonal disruptions, as measured by SMI-31; and the decreased presence of normal, delicate brain vasculature, and the increased presence of simple vascular hyperplasia, as measured by Factor VIII endothelial staining.

## Discussion

This study identified distinctive metabolic profiles for gliomas according to the status of *IDH* mutation, histological subtype, and malignant progression. Several metabolites including those comprising the *in vivo* total choline peak, as well as glutamate and glutamine, were correlated with levels of 2HG and histopathological parameters. The results obtained in this project provide improved characterization of the metabolic pathways in these lesions, and given that many of these metabolites are measurable *in vivo*, may be of use for improving the non-invasive assessment and monitoring of patients.

When considering all histological subtypes, choline-containing compounds were found to be elevated in samples from lesions that had undergone progression. Previous studies in our group have shown increases in PC associated with Grade III and primary Grade IV lesions^13^. These results show that tCho was also elevated and may serve an *in vivo* biomarker for MP. The association of AS lesions with PC and Cho, and OD lesions with GPC may reflect alterations in the phospholipid metabolism found in the choline kinase pathway for individual subtypes. While a general increase of total choline has been associated with higher-grade lesions as well as the OD subtype, the differences in choline-species between distinct histologies was previously unknown and warrants additional investigation.

Increases in Glu and Gln that were associated with progression to GBM may be a product of elevated glutaminolysis, which leads to an additional production of alanine and aspartate^26^. Considering that Glu, Gln, Ala, and Asp were elevated in Grade IV lesions, these metabolites may present another avenue for monitoring patients with MRSI. The recent advent of hyperpolarized ^13^C spectroscopic imaging and successes of many novel hyperpolarized imaging-agents such as [1-^13^C] pyruvate, [1-^13^C] glutamate, and [1-^13^C] α-ketoglutarate may provide additional promising approaches to investigate this pathway, in addition to that of the *IDH* mutation^27^,^28^,^29^.

The production of reactive oxygen species (ROS) promotes genomic instability in tumors and glutathione is one of several molecules involved in eliminating ROS. While its role in brain tumors remains to be elucidated, it appears to play a role in therapeutic sensitivity and resistance^30^. Increased production of GSH in samples evaluated in the current study may be linked to the increased oxidative stress, disregulation of the GSH pathway, or treatment related changes in high-grade lesions. Within the context of *IDH*-mutated gliomas, studies in GBM cell lines have observed a decrease in GSH in cells that artificially overexpress the *IDH* mutation^31^,^32^, which has been hypothesized as a possible mechanism for the radiosensitivity observes in these lesions. However, our results demonstrate a positive correlation between 2HG and GSH levels, suggesting that perhaps there may be alternate cellular mechanisms that require further investigation.

Glycine and other amino-acid metabolites have been previously reported to be elevated in Grade IV lesions^33^, and are consistent with the demands for nucleotide synthesis present in highly proliferative cells. The results of this study corroborate the prior data and further demonstrate significantly elevated Gly in anaplastic oligodendrogliomas. As this metabolite is measurable using *in vivo* techniques, Gly should be explored as a biomarker for MP in olidodendroglioma.

Prior works by our group in a subset of fifty-two patients from this study compared LGG metabolism with that of primary and recurrent GBM^13^, as well as established that the elevation of 2HG in *IDH*-mutated lesions was detectable using magnetic resonance^34^. This latter work was corroborated by others^35^,^36^ and laid the necessary groundwork for this metabolomic HR-MAS investigation between tumors of different *IDH* status and histological subtypes. Given the equilibrium between alpha-ketoglurate and glutamate in the glutamate-glutamine cycle^37^, it has been predicted that there would be a decrease in glutamate in *IDH*-mutated tumors, but this has been difficult to assess using *in vivo* methods, which suffer from significant limitations in resolution and signal-to-noise. The results from our study provide confirmation that there are reductions in glutamate in *IDH*-mutated lesions, as well as reductions in GABA, the production of which is tightly linked with available pools of glutamate and glutamine. When we evaluated the Grade II and Grade III lesions together, we also found elevations in all of the choline-species, including the total choline peak, in *IDH*-mutated tumors.

While the previous comparison characterized differences between wild-type and *IDH*-mutated lesions, we further sought to evaluate *IDH*-mutated tumors specifically and the correlation between levels of 2HG and other metabolites and histopathology features. Within *IDH*-mutated tumors, we found many metabolites including glutamate and GABA that were positively correlated with increased 2HG levels. In order to reduce any potentially confounding effects created by differences in tumor cellularity, we normalized these levels by average cell density. To our surprise, these results held significant, therefore the metabolite differences observed cannot be explained by differences in tumor cellularity alone. Interestingly, the correlation between 2HG levels and mitotic activity, axonal disruption, and neovascularization, further suggestive that high levels of 2HG may be associated with malignant progression, and that these cells could be sensitive to therapeutics targeted to these properties.

It is important to highlight that there have been previous *in vivo* studies that have investigated the relationships between *IDH* mutation and cellularity, which have yielded similar results. Specifically, the results of our project corroborated the *in vivo* study by Pope *et al*.^38^, which found elevations in total choline and mitotic activity in *IDH*-mutated tumors. The results obtained by De La Fuente, *et al*.^39^, however, recently reported correlation between 2HG levels and cell density, but not mitosis. This may be explained by the decreased sensitivity of *in vivo* techniques in detecting 2HG in small to medium sized lesions, and highlights the gains in sensitivity associated with high-resolution NMR. These results suggest increased cellular pathology associated with 2HG production and that total choline may serve as a promising *in vivo* biomarker for assessing the increased cellularity associated with *IDH* mutation.

Owing to the rarity of *IDH2* mutations, which occur in approximately 3% of diffuse gliomas^40^, this study has treated the various mutated residues of *IDH1* and *IDH2* collectively. In 2011, Reitman *et al*. established that a majority of the metabolic alterations of *IDH* mutations were shared across mutations in *IDH1* and *IDH2* when compared with wild-type gliomas^31^. Subsequent *in vitro* work has revealed differential 2HG production based on the subcellular localization of IDH2 enzymes in the mitochondria, providing evidence that *IDH2* mutations are robust to the absence of the wild-type enzyme while *IDH1* mutations are not^41^. Although limited in sample size, a recent *in vivo* study at ultra-high field strength supports this result by demonstrating an over three-fold increase in 2HG levels in *IDH2*-mutated lesions^42^,^43^,^44^,^45^. Taken together, these studies support further investigation into the metabolic differences between *IDH1* and *IDH2* mutations that are downstream of 2HG.

There is a clear need for diagnostic and treatment biomarkers for the IDH-pathway that can keep pace with the development of novel therapies. While measuring 2HG using *in vivo* spectroscopic techniques would likely provide the most specific marker for these lesions, the clinical development and determination of the optimal sequence is still under active investigation. While the approaches presented thus far are promising, achieving the necessary spatial resolution and signal-to-noise for defining tumor margins, as well as reliably resolving 2HG from overlapping metabolites, such as GABA, remains challenging. Given the strong correlations of levels of 2HG with levels of metabolites such as choline, creatine and glutamate, which are routinely measured using *in vivo*^1^H MRSI and are present in higher concentrations, a combined approach may be valuable for evaluating treatment effects.

The ultimate goal of this work is to improve the clinical management of patients with glioma. The spectral profiles obtained in this study may aid in developing non-invasive MRSI methods to better diagnose and monitor patients based on underlying tumor metabolism, and further characterize the *IDH*-mutated molecular subtype. Most importantly, we hope that this study will lead to improved outcome and quality of life for these patients.

## Additional Information

**How to cite this article**: Jalbert, L. E. *et al*. Metabolic Profiling of *IDH* Mutation and Malignant Progression in Infiltrating Glioma. *Sci. Rep.* **7**, 44792; doi: 10.1038/srep44792 (2017).

**Publisher's note:** Springer Nature remains neutral with regard to jurisdictional claims in published maps and institutional affiliations.

## Supplementary Material

Supplementary Materials

## Figures and Tables

**Table 1:** Patient and tissue sample population by grade at the time of surgery, histological subtype, and *IDH* mutation status.

Grade/Hist	Total patients (samples)	*IDH*-wt (samples)	*IDH-*mut (samples)	*IDH*-uncertain (samples)
All	126 (219)	15 (26)	108 (190)	3 (3)
II	62 (103)	10 (16)	50 (85)	2 (2)
III	50 (89)	2 (4)	47 (84)	1 (1)
IV	14 (27)	3 (6)	11 (21)	—
AS	52 (83)	8 (12)	42 (69)	2 (2)
OA	30 (57)	5 (10)	24 (46)	1 (1)
OD	44 (79)	2 (4)	42 (75)	—

The patient population comprised astrocytoma, oligodendroglioma, and oligoastrocytoma histological subtypes. The majority of patients (88%) harbored *IDH*-mutant lesions and 64 patients (51%) had undergone MP. It is of note that 10 of the patients were scanned at two distinct recurrences.

**Table 2:** Statistically significant metabolite levels of all histologies between *IDH* status and tumor grade.

	All histologies	(med ± SE)		All histologies	(med ± SE)
Metabolite	*IDH*-mut	*IDH*-wt	Grade II	Grade III	GBM
Cho Cho	7.4 ± 1.26	3.72 ± 0.73	—	—	—
GPC	20.2 ± 3.64	9.2 ± 2.09	—	—	—
PC	11.54 ± 3.0	6.58 ± 1.37	9.38 ± 1.68	14.26 ± 5.04	26.44 ± 15.08
tCho	38.18 ± 5.81	21.03 ± 3.16	23.05 ± 5.01	36.31 ± 9.37	64.27 ± 18.48
GSH	—	—	11.52 ± 1.54	12.76 ± 4.51	17.28 ± 7.06
hTau	—	—	2.05 ± 0.63	—	4.07 ± 0.77
Tau	—	—	14.87 ± 2.3	31 ± 4.24	29.6 ± 10.1
2HG	8.17 ± 1.01	2.82 ± 0.47	5.43 ± 1.1	7.88 ± 1.57	13.7 ± 2.57
Glu	49.84 ± 4.99	94.18 ± 33.03	41.76 ± 6.17	52.18 ± 8.13	79.79 ± 24.65
Gln	—	—	33.09 ± 5.64	—	38.24 ± 15.39
Glc	—	—	31.31 ± 5.12	—	35.74 ± 44.41
Ala	—	—	11.73 ± 2.31	17.12 ± 9.49	20.87 ± 10.88
Asp	—	—	37.83 ± 4.89	39.33 ± 5.83	71.32 ± 27.7
GABA	2.92 ± 0.42	6.48 ± 3.04	—	—	—
Gly	—	—	33.46 ± 5.67	46.12 ± 8.57	78.03 ± 29.75
Bet	—	—	0.85 ± 0.1	0.88 ± 0.18	1.27 ± 0.6

Mixed-effects logistical regression results demonstrated significant differences in various metabolite levels between *IDH* genotype and tumor grades (*p* < 0.05). Metabolite levels are presented for statistically significant metabolites at a tissue sample level. These results are presented as median spectral areas for each metabolite as fit by HR-QUEST.

**Table 3:** Metabolite and histopathology correlations with 2HG levels.

Variable correlated	Correlation type	Number of pairs	*p* value
Cho	+	62	*p* < 0.001
GPC	+	70	*p* < 0.001
PC	+	60	*p* < 0.001
tCho	+	70	*p* < 0.001
PE	+	58	*p* < 0.001
GSH	+	43	*p* < 0.001
Tau	+	30	*p* < 0.001
Glu	+	66	*p* < 0.001
Gln	+	62	*p* < 0.001
Asp	+	46	*p* < 0.001
myo-I	+	64	*p* < 0.001
SI	+	39	*p* < 0.001
GABA	+	35	*p* = 0.001
PCr/Cr	+	69	*p* < 0.001
Gly	+	55	*p* < 0.001
Bet	+	45	*p* < 0.001
Tbr	+	48	*p* < 0.001
Mitosis (MIB1)	+	55	*p* = 0.016
Axonal disruption (SMI31)	+	53	*p* = 0.032
Simple vascular neoplasia	+	49	*p* = 0.01
Complex vascular neoplasia	+	54	*p* = 0.047

Each of the study parameters correlated with 2HG is presented as determined from Pearson testing for all continuous variables and Kendell Tau testing for ordinal variables. Here we present the correlated parameters with their associated number of tested pairs and median *p* values. Normalization by cell density did not affect any correlation within *IDH*-mutant tumors.
