# Supplementary material for: Metabolic Profiling of IDH Mutation and Malignant Progression in Infiltrating Glioma
Source: Sci Rep. 2017 Mar 22;7:44792. doi: 10.1038/srep44792 (PMC5361089; doi:10.1038/srep44792)
Supplement: Supplementary Materials [file srep44792-s1.pdf]

# Supplementary Materials

## Metabolic Profiling of *IDH* Mutation and Malignant Progression in Infiltrating Glioma

Llewellyn E. Jalbert, Adam Elkhaled, Joanna J. Phillips, Evan Neill, Aurelia Williams, Jason C. Crane, Marram P. Olson, Annette M. Molinaro, Mitchel S. Berger, John Kurhanewicz, Sabrina M. Ronen, Susan M. Chang, and Sarah J. Nelson

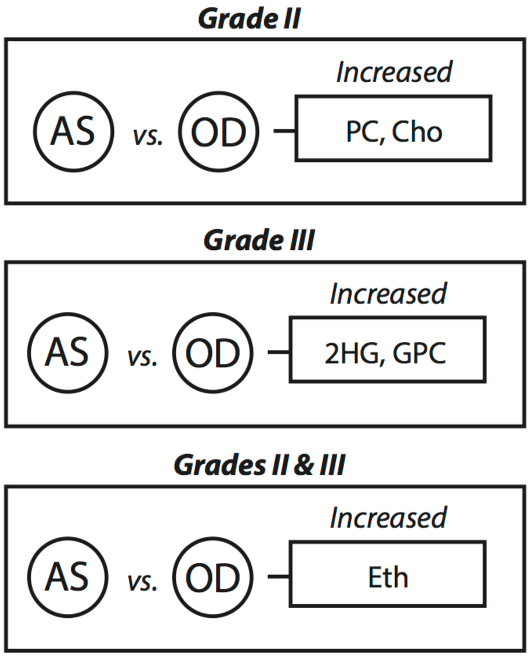


**Supplementary Figure S1. Flow diagram of metabolite differences between astrocytoma and oligodendroglioma subtypes within histological grades.** When compared within each histological grade, we observed several metabolites that were elevated in oligodendroglial lesions when compared with astrocytomas. Oligoastrocytomas were excluded from this comparison. These included PC and Cho (Grade II), 2HG and GPC (Grade III), and Eth (Grades II and III combined).

| | | AS | (med ± SE) | OD | (med ± SE) |
|---|---|---|---|---|---|
| **Metabolite** | **Grade II** | **Grade III** | **GBM** | **Grade II** | **Grade III** |
| **Cho** | 3.65 ± 0.81 | 8.96 ± 2.36 | 16.59 ± 3.69 | - | - |
| **GPC** | - | - | - | 16.43 ± 3.16 | 41.77 ± 12.27 |
| **PC** | 4.18 ± 1.26 | 13.73 ± 6.99 | 28.66 ± 13.14 | - | - |
| **tCho** | 12.26 ± 6.32 | | 65.34 ± 17.1 | 29.34 ± 6.2 | 83.83 ± 19.58 |
| **PE** | 12.21 ± 5.44 | 36.61 ± 8.54 | - | - | - |
| **Tau** | - | - | - | 20.82 ± 2.38 | 35.98 ± 5.09 |
| **2HG** | 4.3 ± 1.55 | - | 11.96 ± 2.63 | 5.45 ± 1.26 | 15.2 ± 2.8 |
| **Glu** | 21.47 ± 16.42 | - | 82.37 ± 24.09 | 37.87 ± 9.39 | 71.05 ± 15.67 |
| **Gln** | 19.32 ± 6.08 | 46.5 ± 8.58 | - | - | - |
| **Gly** | - | - | - | 36.68 ± 5.08 | 52.01 ± 15.59 |
| **Bet** | - | - | - | 0.91 ± 0.12 | 2.17 ± 0.24 |

**Supplementary Table S1. Statistically significant metabolite levels associated with malignant progression within AS and OD histological subtypes.** Mixed-effects logistical regression results demonstrated significant differences in various metabolite levels associated with malignant progression ($p < 0.05$). Metabolite levels are presented for statistically significant metabolites at a tissue sample level. These results are presented as median values of the normalized areas under the curve as quantified by HR-QUEST.

## Supplementary Methods

### *Histopathology analysi*s

Slides stained with H&E, MIB-1, and *IDH1*R132HR132H were scored to assess histological characteristics. For the H&E-stained slides, a tumor score was given on the basis of the contribution of tumor cellularity to total cellularity. A score of 0 denoted neuropil without tumor; 1 indicated an infiltrating tumor margin containing detectable but not abundant numbers of tumor cells; 2 denoted a more cellular-infiltrated zone; 3 denoted highly cellular tumor with few non-neoplastic cells. Tissue samples with a tumor score of 0 were excluded from analysis. Total cell-density was also determined as an average number of cells per 200x field. For MIB-1-stained slides, a labeling index [(MIB-1-positive nuclei per total tumor cells counted per 200x field) x 100%] was calculated based on the evaluation of at least three fields and >1000 cells. Digital images were captured using a microscope (Olympus, Model BX41TF) and digital camera (Olympus, Model DP70).

For SMI31- stained slides, a score was assigned to each slide on the basis of the extent of disruption of the normal axonal architecture. A score of 0 denoted no disruption; 1 denoted minimal disruption; 2 denoted mild disruption; 3 denoted severe disruption with no detectable SMI-31-positive processes. Microvascular morphology was graded based upon H&E staining and Factor VIII immunohistochemistry as delicate (resembling normal cerebral vessels), simple vascular hyperplasia (circumferential hyperplasia with definitive lumen), or complex microvascular hyperplasia (glomeruloid-type vessels). An overall score for each vascular component (delicate, simple vascular hyperplasia, and complex microvascular hyperplasia) based on Factor VIII was derived from its relative contribution to total vascularity using a four-tier ordinal scale (0, no contribution; 1, minimal; 2, prevalent; 3, extensive) at a magnification of 200x.
